# Advances of Hyaluronic Acid Nasal Injection Techniques and Complications: A Systematic Review

**DOI:** 10.1007/s00266-025-05194-z

**Published:** 2025-09-02

**Authors:** Yilei Ma, Mengying Jin, Yonghuan Zhen, Yang An

**Affiliations:** Department of Plastic Surgery, Peking University Third Hospital, No. 49 North Garden Road, Haidian District, Beijing, 100191 China

**Keywords:** Hyaluronic acid, Non-surgical rhinoplasty, Systematic review, Injection techniques, Complication

## Abstract

**Background:**

The increased demand for non-surgical rhinoplasty using hyaluronic acid (HA) has given impetus to explore more injection techniques and has also placed a higher demand on the coping of complications.

**Objectives:**

The purpose of this systematic review is to provide clinicians with recommendations by broadly consolidating data on HA injections for rhinoplasty injection techniques and complications, and presenting the advantages and disadvantages of each technique and the treatment of complications.

**Methods:**

A systematic electronic literature search using keywords and MESH search terms over the PubMed/Medline, Cochrane Central, Embase, Web of Science, and CNKI online databases was conducted from 2000 to 2024. Risk of bias assessment was performed for all included articles.

**Results:**

There were 37 papers with a total of 7339 patients included in the study. Four different injection regimens were summarized. The incidence of serious complications such as blindness, skin necrosis, and cerebral infarction was only 3‰. Mild complications including bruising, hematoma, edema, and asymmetry had a higher incidence of 13.34%.

**Conclusion:**

Existing techniques for hyaluronic acid nasal injections can be summarized as top-to-bottom approach, bottom-up approach, critical site injections, and multi-plane injections, with the latter two more innovative injections reporting higher complication rates in comparison.

**Level of Evidence I:**

This journal requires that authors assign a level of evidence to each article. For a full description of these Evidence-Based Medicine ratings, please refer to the Table of Contents or the online Instructions to Authors  www.springer.com/00266

**Supplementary Information:**

The online version contains supplementary material available at 10.1007/s00266-025-05194-z.

## Introduction

In recent years, there has been a significant rise in the demand for all types of plastic surgery programs [1]. As an important component of facial esthetics, the increase in rhinoplasty reached 21.6% in 2023 [1], in which hyaluronic acid injections for rhinoplasty have been widely used in clinical practice due to their advantages of low trauma, fast recovery, reduced patient downtime, relatively low price, and high levels of reversibility through the use of hyaluronidase. This technique is often appropriate for patients with certain defects in nasal tip and dorsal morphology, including insufficient projection (i.e., a need to raise the nasal tip), a nasal hump, and asymmetry [2]. In these patients, the use of hyaluronic acid to enhance the nasal root and base, to adjust the nasal tip, and to correct minor deformities [3] can achieve results similar to those of surgical rhinoplasty at a much lower cost. Rhinofilling can also be performed in place of augmentation rhinoplasty in certain patients with moderate deformities, including an acute nasolabial angle, nasal dorsum projection, nasal frontal angle depression, nasal columella protrusion, and/or nasal tip ptosis [3]. Patients undergoing minor corrections and improvements after surgical rhinoplasty have had good results with HA nasal injections.

As a result of the increase in the volume of injections, the injection technique of hyaluronic acid rhinoplasty has undergone some research. Basically, the consensus injection technique is along the nasal midline, starting from the nasal root to the nasal tip [4], injecting under low pressure, with the injection plane to the periosteal or cartilaginous membrane plane [5] to avoid vascular complications. The adjusted sites are mainly the nasal root, nasal dorsum, nasal tip, nasal wings, and subnasal, and the total injection volume is mostly around 1 ml. However, there is still a relative lack of standardized injection techniques, with overcorrection/undercorrection and inaccurate injection depth [6], and thus, some clinicians have proposed more innovative but less systematically practiced injection protocols, such as five-point liquid rhinoplasty and other protocols [7]. The effect of injection tools, mainly the difference of needles and cannulas on injection, has also been explored. Necessary preparations before injection, such as the use of local anesthetics and epinephrine, constrict the vasculature and may be associated with a reduced risk of embolism [8].

With the current surge in injections for hyaluronic acid rhinoplasty, concerns about complications are even more critical. Most clinicians recognize the low risk of hyaluronic acid injections, but serious complications continue to be reported in practice, mostly related to the complex blood supply to the nose. There are two main blood supplies to the nose, one is the facial artery from the external carotid artery, which extends through the corners of the mouth and the lateral side of the nose to the medial canthus renamed the medial canthal artery, and the other is the dorsal nasolabial artery, the terminal branch of the ophthalmic artery from the internal carotid artery. These blood vessels, which show complex intertwining and anastomosis, almost always travel in the more superficial areas, i.e., between the superficial fat layer and the fibromuscular layer, which forms the structural basis for the occurrence of vascular complications. Serious vascular complications include blindness [9–11], cerebral infarction [10], skin necrosis [12], and motor nerve palsy [13]. In addition to this, early complications include erythema, ecchymosis, and mass formation, and late complications include granuloma formation, delayed hypersensitivity reaction, filler migration, and zygomatic edema [14]. This article was conducted with an aim to provide evidence regarding the techniques and complications of hyaluronic acid nasal injections to guide clinicians in evidence-based practice.

## Methods

### Search Strategy

A comprehensive literature search was carried out in PubMed, Embase, Web of Science, Cochrane, and CNKI to collect the relevant literature. The search terms were mainly "rhinoplasty" and "hyaluronic acid," comprising of headings of ("rhinoplasty"[MeSH Terms] OR "rhinoplasty"[All Fields]) AND ("hyaluronic acid"[MeSH Terms] OR ("hyaluronic"[All Fields] AND "acid"[All Fields]) OR "hyaluronic acid"[All Fields]).

The literature was included in this systematic review from 2000. There were no language restrictions. Where applicable, relevant literature was translated into English. If full publications were not available online, they were requested (if possible) from the first author.

### Inclusion and Exclusion Criteria

**Fig. 1:**
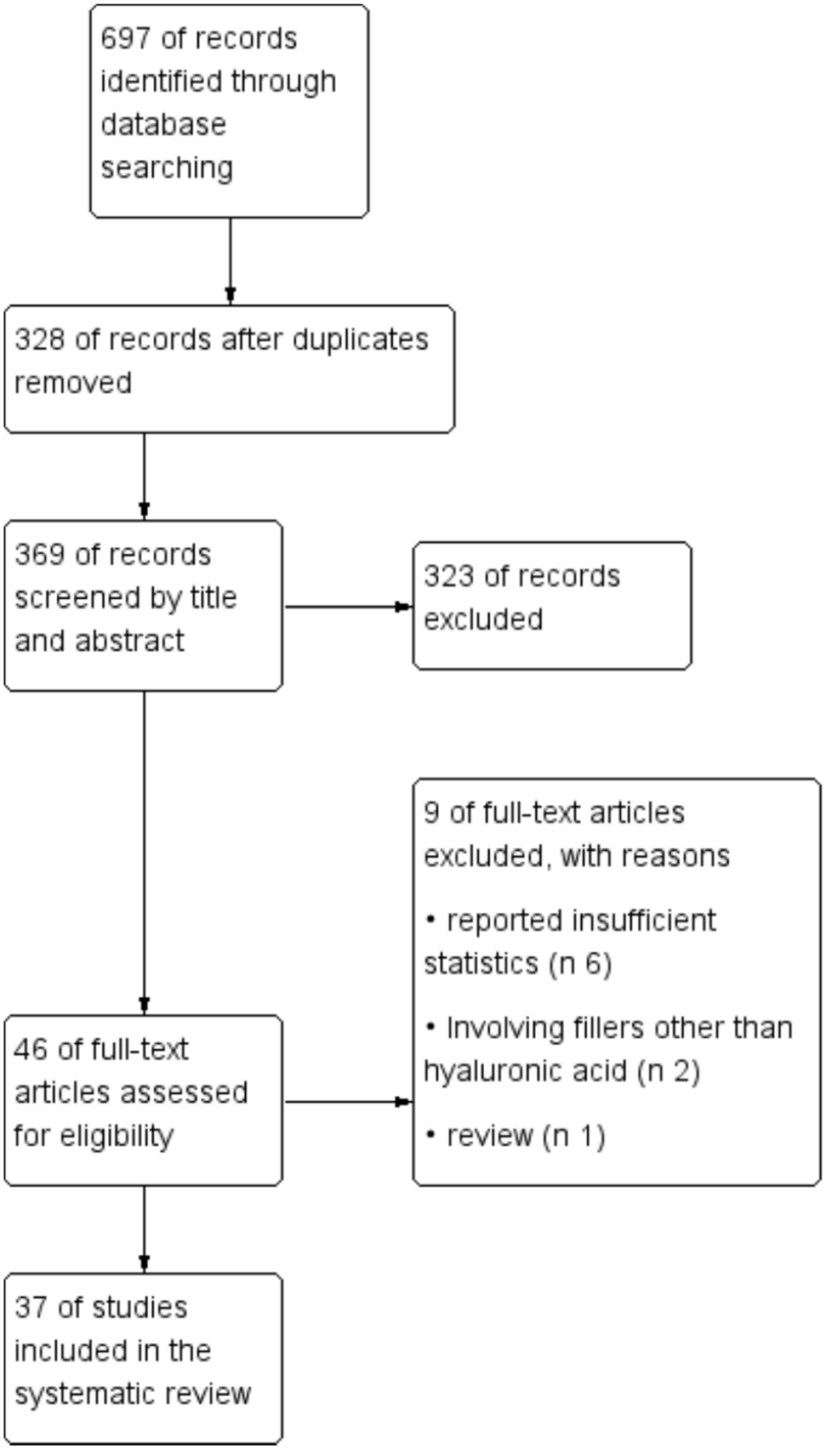
Flow diagram showing the process of screening

After getting the retrieved literature, the duplicates were sifted out, and then, the title and abstract were read to select the relevant literature. The screened literature was intensively read in its entirety, and final inclusion was determined based on inclusion and exclusion criteria (Fig. 1).

The literature included in this paper consists mainly of Retrospective literature, Case reports, Randomized Controlled Trials, and Non-Randomized Trials. Narrative Reviews, Systematic Reviews, Technical Reports, Expert Opinions, Descriptive studies, etc., were excluded from the section on data collation regarding complication rates. Information about the excluded 14 papers was presented in Supplementary Table 1. Integration was performed for the data sources used to calculate complication rates. A summary of study characteristics that were included is illustrated in Table 1, which includes authors; no. of patients; age and gender of the patients; fillers used; and follow-up period. The included papers all had research centered on injection technique or injection complications, or at least met the requirement of demonstrating adequate attention to one point.

**Table 1:** Characteristics of the studies included in the review

Type of work	No.	References	Year	No. of patients	Age	M/F	Fillers used	Follow-up period
RCT	1	Li et al. [15]	2022	157	AVG = 31.3	10/147	VYC-20L	Weeks 8, 16, and 24 after the last treatment in the control period and at weeks 36 and 48 in the treatment group
	2	Wang et al. [16]	2022	131	20–60 (AVG = 34.7)	4/127	Restylane Lyft (Galderma, Uppsala, Sweden)	12 months
Prospective	3	Liew et al. [17]	2016	29	20–61 (AVG = 40.2)	3/26	Juvéderm VOLUMA [Allergan plc, Dublin, Ireland] with lidocaine injectable gel	Followed for a mean of 378 days (range 204–434 days)
	4	Nikolis et al. [18]	2023	33	19–57 (AVG = 33.21)	0/33	Restylane® Lyft (HA-L, Galderma, Uppsala, Sweden)	6–8 months
	5	Santorelli et al. [19]	2020	62	17–68 (AVG = 29)	5/57	Either VYC-20 (Voluma^®^; 20 mg/mL HA) or VYC-17.5 (Volift^®^; 17.5 mg/mL HA)	> 12 months
	6	Liapakis et al. [20]	2013	11	NA	NA	HA	> 12 months
Retrospective	7	Bektas et al. [21]	2020	62	20–52 (AVG = 30.5)	8/54	1 of 3 brands of HA filler (Allergan, Dublin, Ireland; Merz Aesthetics, Raleigh, NC; Neauvia, Lugano, Switzerland)	Between 6 and 24 months (median 11.2 months)
	8	Bertossi et al. [22]	2021	61	19–54 (AVG = 32)	16/45	The high-G’ and high cohesivity dermal filler, VYC-25L (25 mg/mL HA with 0.3% lidocaine; Volux), from the Vycross range (Allergan, Dublin, Ireland)	6–9 months
	9	Chen et al. [23]	2020	198	18–53 (AVG = 30.4)	9/189	HA	6 months after the injection
	10	Giammarioli et al. [24]	2023	101	95% CI = 28.3–38.9	10/91	A 25 mg/mL HA filler (Teosyal Ultra Deep™, Teoxane Switzerland) with lidocaine 0.3%	1 year
	11	Harb et al. [25]	2024	487	18–70 (Median = 27)	7/480	Cross-linked hyaluronic acid gels (Teosyal Puresense Ultra Deep 25 mg/mL with lidocaine [Teoxane Laboratories, Geneva, Switzerland] and Belotero Intense 25.5 mg/mL with lidocaine [Merz Pharma, Frankfurt, Germany])	> 2 weeks
	12	Harb et al. [26]	2020	5000	18–78 (AVG = 27)	298/4702	Cross-linked hyaluronic acid gels [Teosyal Ultra Deep 25 mg/ml integral lidocaine (Teoxane Laboratories, Geneva, Switzerland); Perfectha Subskin 20 mg/ml (Sinclair Pharma, London, United Kingdom)]	4–6 weeks
	13	Jalali [27]	2024	492	18–69 (AVG = 30)	25/467	VYC-25L (Juvéderm® Volux™ XC, Allergan Aesthetics, an AbbVie Company)	The mean duration of follow-up was 11.1 months.
	14	Rauso et al. [28]	2017	52	18–61 (AVG = 29.7)	9/43	a 20-mg/mL smooth, cohesive, and viscous HA filler (Juvederm Voluma; Allergan plc, Dublin, Ireland)	15 days
	15	Segreto et al. [29]	2019	70	AVG = 27	9/61	Juvederm 4 (Allergan plc, Dublin, Ireland)	12 months
	16	Turk et al. [30]	2024	40	18–62 (AVG = 36.1)	3/37	An HA filler containing 20mg/mL HA (Restylane®-lidocaine; Galderma, Lausanne, Switzerland)	6 months
	17	Yordanov et al. [31]	2019	11	27–42 (AVG = 33.82)	3/8	Restylane (Q-Med, Uppsala, Sweden) – a high-G’product (512Pa) with HA concentration of 20 mg/ml and lidocaine hydrochloride of 3 mg/ml	12 months
	18	Romeo [32]	2023	206	18–65 (AVG = 40)	11/195	An LG Chem Ltd., Korea medium density (2.0%–2.2% of HA) cross-linked gel filler	> 6 months
	19	Jung [33]	2019	96	22–48 (AVG = 33.8)	7/89	Hyaluronic acid filler (Teosyal® Puresense Ultra Deep, Teoxane Lab, Switzerland)	3 months
	20	Josipovic [7]	2021	20	23–62 (AVG = 37.8)	5/15	Juvéderm Voluma (*n* = 13), Juvéderm Volux (*n* = 6), or Belotero Volume (*n* = 1)	6 months
Case report	21	Benjamin et al. [34]	2020	1	28	0/1	Juvederm (hyaluronic acid)	4 days
	22	Chen et al. [12]	2016	1	32	0/1	HA	48h
	23	Chen et al. [13]	2016	1	22	0/1	HA	14 days
	24	Eldweik [11]	2021	1	32	0/1	HA	8 weeks
	25	Fan et al. [35]	2016	2	28, 24	0/2	HA	1 month
	26	Kim et al. [36]	2014	1	23	1/0	HA (Restylane R; Q-Med AB, Uppsala, Sweden)	3 months
	27	Kim et al. [37]	2014	1	NA	0/1	HA	6 months
	28	Lee et al. [38]	2017	1	25	0/1	HA (Bellast, Dongkook Pharm. Co., Seoul, Korea)	6 months
	29	Leupe et al. [39]	2016	2	31, 19	2/0	HA	More than 3 years; 1 year
	30	Lucaciu et al. [40]	2022	2	43, 29	0/2	HA	2 months; 48 days
	31	Sheptulin et al. [41]	2022	1	40	0/1	HA (Juvéderm Voluma, Allergan, CA, USA)	3 months
	32	Souza et al. [42]	2021	1	29	0/1	HA	More than 1 week
	33	Yu et al. [10]	2023	1	19	0/1	HA	2 years after injection
	34	de Lacerda et al. [43]	2007	1	52	0/1	Hyaluronic acid (Voluma, Corneal, Paris, France)	12 months
	35	Kim et al. [44]	2013	1	NA	0/1	HA	6 months
	36	Kim et al. [45]	2011	1	30	0/1	Hyaluronic acid (Restylane R, Q-Med AB, Uppsala, Sweden)	6 months
	37	Piggott et al. [46]	2011	1	18	1/0	HA	NA
	Total		7339					

### Risk of Bias Assessment

**Fig. 2:**
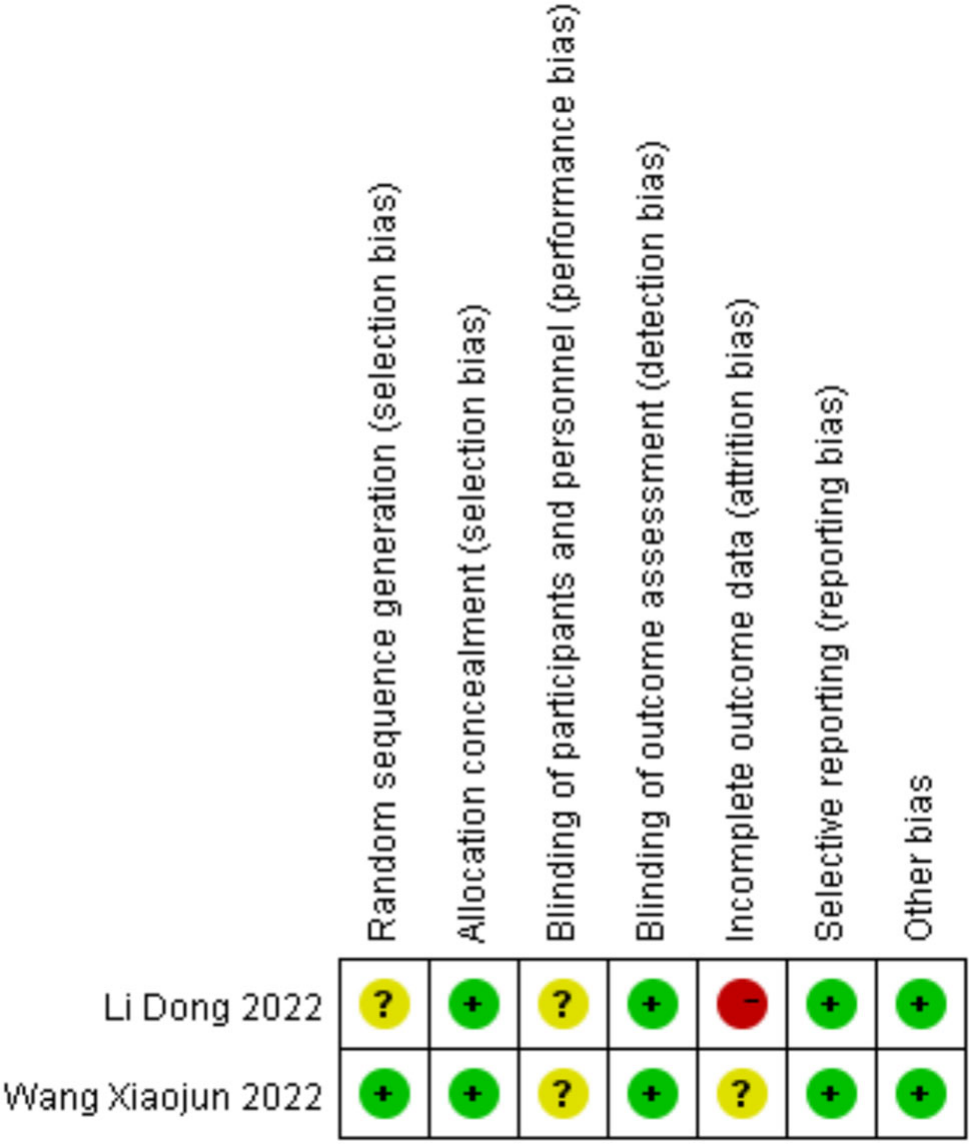
Risk of bias assessment (RCT)

**Fig. 3:**
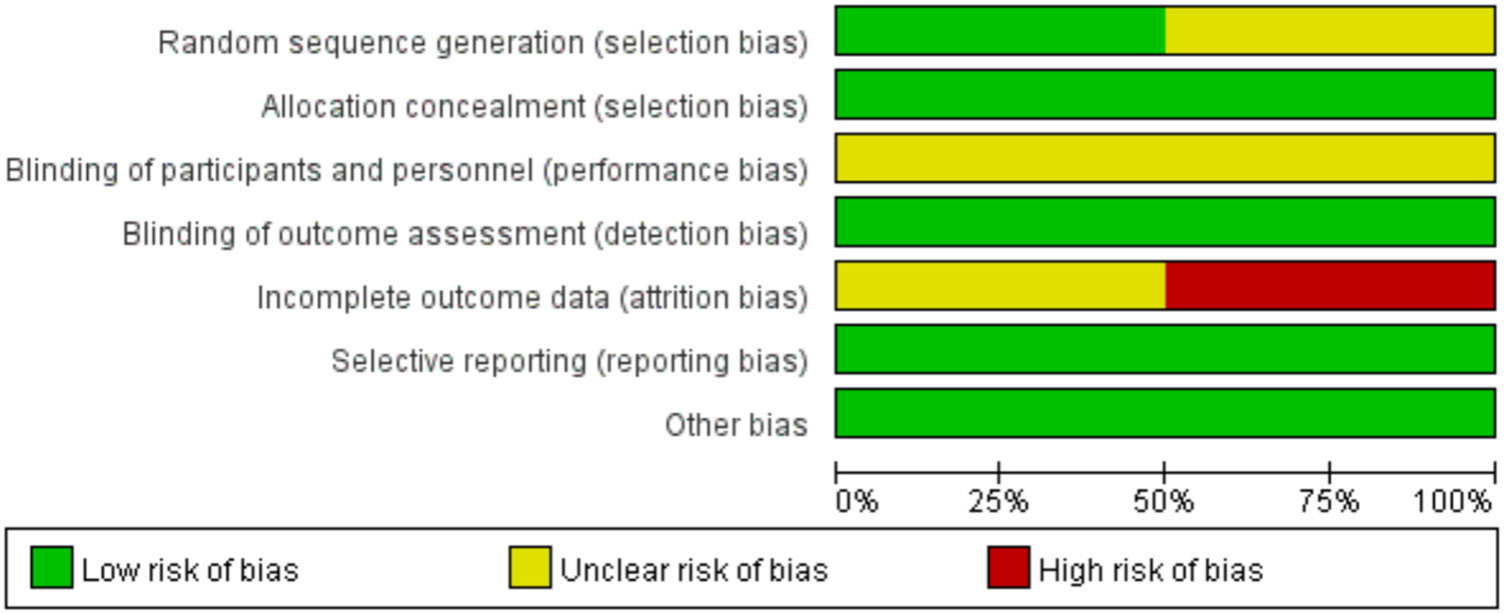
Risk of bias assessment (RCT)

Regarding RCTs, the Cochrane risk of bias tool was used for quality evaluation [47] (Figs. 2 and 3). For non-randomized trials, the NOS scale was utilized in the literature quality assessment (Table 2). Specific rating explanations are presented in Supplementary Table 2.

**Table 2:** The NOS scale

Domains	Leading explanatory questions	Bektas et al. [21]	Bertossi et al. [22]	Chen et al. [23]	Giammarioli and Liberti [24]	Harb and Abdul-Razzak [25]	Harb and Brewster [26]	Jalali [27]	Liew et al. [17]	Nikolis et al. [18]	Rauso et al. [28]	Santorelli and Marlino [19]
Selection	Representativeness of the exposed cohort	☆	☆	☆	☆	☆	☆	☆	☆	☆	☆	☆
	Selection of the non-exposed cohort	☆	☆	☆	☆	☆	☆	☆	☆	☆	☆	☆
	Ascertainment of exposure	N	N	N	N	N	N	N	N	N	N	N
	Demonstration that outcome of interest was not present at start of study	☆	☆	☆	☆	☆	☆	☆	☆	☆	☆	☆
Comparability	Comparability of cohorts on the basis of the design or analysis	☆	☆	☆	☆	☆	☆	☆	☆	☆	☆	☆
Outcome	Assessment of outcome	☆	☆	☆	☆	☆	☆	☆	☆	☆	☆	☆
	Was follow-up long enough for outcomes to occur	☆	☆	☆	☆	N	N	N	☆	☆	N	☆
	Adequacy of follow-up of cohorts	☆	☆	☆	☆	N	N	☆	☆	☆	☆	☆
	Scores	7	7	7	7	5	5	6	7	7	6	7

Domains	Leading explanatory questions	Liapakis [20]	Segreto [29]	Turk [30]	Yordanov and Shef [31]	Romeo [32]	Jung [33]	Josipovic et al. [7]
Selection	Representativeness of the exposed cohort	☆	☆	☆	☆	☆	☆	☆
	Selection of the non-exposed cohort	☆	☆	☆	☆	☆	☆	☆
	Ascertainment of exposure	N	N	N	N	N	N	N
	Demonstration that outcome of interest was not present at start of study	☆	☆	☆	☆	☆	☆	☆
Comparability Outcome	Comparability of cohorts on the basis of the design or analysis	☆	☆	☆	☆	☆	☆	☆
	Assessment of outcome	☆	☆	☆	☆	☆	☆	☆
	Was follow-up long enough for outcomes to occur	☆	☆	☆	☆	☆	N	☆
	Adequacy of follow-up of cohorts	☆	☆	☆	☆	☆	☆	☆
	Scores	7	7	7	7	7	6	7

## Results

### Injection Techniques

Table 3 gives the injection techniques included in the study and their associated ethnographic information, pros and cons, complications, and satisfaction.

**Table 3:** Injection techniques

Technique	Plane	Site	Patients	Advantage	Disadvantage	Complication	Satisfaction
The "top-to-bottom" approach [5, 26]	The supraperiosteal or supracartilaginous planes	(along the middle line of the nose) Nasal root→dorsum→tip	Caucasian; Asian	Minimizing the risk of serious complications	Inability to perform corrections aimed at reducing bone, cartilage, or soft-tissue structure (a common problem with liquid rhinoplasty); undercorrection occurs in a certain percentage of patients	In a retrospective study of 5000 patients, only 1% developed erythema and 3% developed swelling. Infection and skin necrosis rates were 0.04% and 0.06%, respectively.	Only 3 of 5000 (0.06%) patients reported significant dissatisfaction with results.
The "bottom-up" approach [23, 28]	The subperiosteal or supracartilaginous planes	Anterior nasal spine→columella→tip→dorsum+nasal root	Chinese; Caucasian	Minimize the amount of filler injected into the nasal root; correcting the sharp nasolabial angle and increasing the support of the basal columella can help facilitate elevation of the nasal tip.	Nasal tip injections carry a high risk of vascular complications and require strict control of injection volume	1.2% (4/198+148) of patients developed local subcutaneous congestion and bruising after injection, which disappeared within 2 weeks. None of the patients experienced complications such as infection, embolism, necrosis, nasal back light transmission, and widened nasal roots. 0.3% (1/198+148) of patient experienced vascular impairment involving the left ala and the mid-third of the vault on the left side. This patient was treated with hyaluronidase, and the area was completely healed by 1 week. In 2 (/198+148) other patients, transient blanching of the skin developed at the tip of the nose after the peak injection volume; this effect self-resolved in less than 5 min.	Patient satisfaction was evaluated on a visual analog scale (VAS) in which 0 represented the worst possible esthetic outcome and 100 were the best. 84.5% (125/148) of patients indicated a score of >90.
The multi-plane injection technique [23, 32, 33]	The dermal plane	Four soft-tissue areas are monitored: Nasion (P1), Nasal dorsum (P2), Nasal tip (P3), and Subnasal (P4). For the P1, P2, and P3 regions usually, two injections are performed at each side of the nose for a total of seven points.	Caucasian	Better appearance and less filler transfer	Increased risk of serious vascular complications	Minor erythema was seen in 5% (14/280) of the patients, all disappearing after 20 days of using zinc oxide cream. No serious complications.	Under the different injection regimens, patients reported a mean score of 4.7 on a five-point scale and 78% satisfaction, respectively.
	The superficial fatty layer+ the deep fatty layer	Fill the dorsum of the nose from the root, and finally, fill the nasal columella and nasolabial folds.	Korean			No serious adverse events occurred. Temporary mild erythema in 2/96 (2.1%) patients and mild ecchymosis in two patients after treatment. Complications subsided after 3 days and healed without sequelae.	
	Supraperiosteal + subcutaneous injection	Fill the anterior nasal spines and nasal columella first, then fill the dorsum and root of the nose.	Chinese			No serious adverse events occurred. A few patients experienced bruising and swelling.	
The critical site injections [6, 7]	The supraperiosteal or supracartilaginous planes	Standardized to five injection sites: nasal tip, nasal root, cartilaginous dorsum, subnasal, and nasal alar.	Caucasian	Enhance patient safety and improve treatment consistency	Some overfilling occurs, which can be easily resolved by removing excess volume with a needle	Two patients (10%) experienced hematoma requiring no active treatment. There were no instances of bruising, bumps, infection, or skin necrosis.	19 patients (95%) said they were “very satisfied.”
		Standardize into four injection sites: dorsal 1, 2, 3; paramedian 1, 2, 3 (right and left); Tip 1, 2, 3, 4 (adding right and left tp4); endonasal (right and left), a total of 16 points.	Caucasian and Mediterranean descent				

**Fig. 4:**
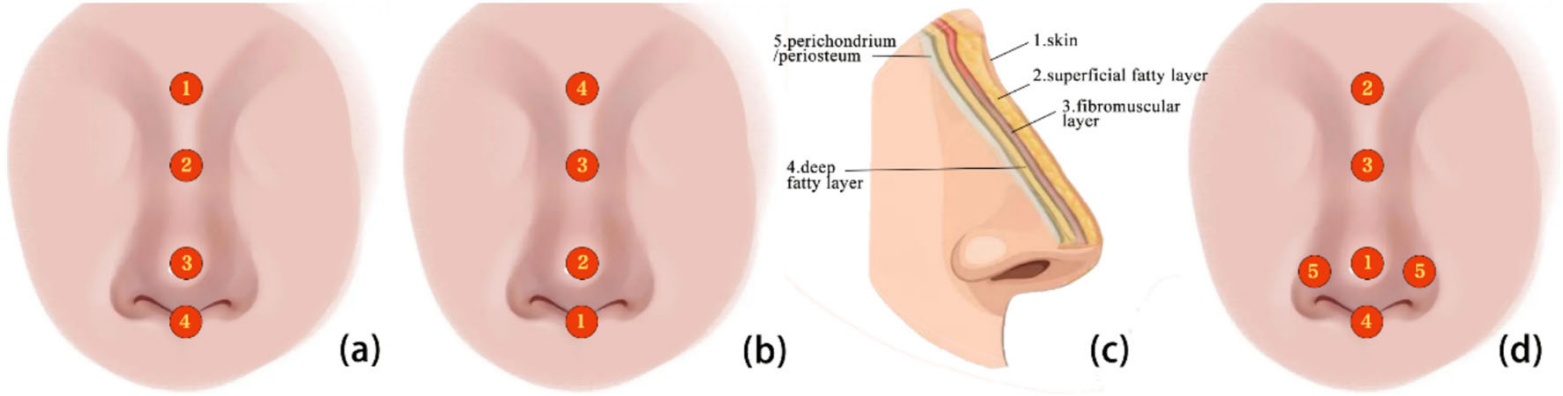
Different sequences and layers of injection options. The “top-to-bottom” approach (**a**); the “bottom-up” approach (**b**); the “multi-plane” injection (**c**); and the “critical site” injection (taking the “Five-point liquid rhinoplasty” as an example) (**d**)

After consolidation, this paper reports four different injection ideas containing seven injection regimens. The difference lies in the order and layers of the injections (Fig. 4). The two more popular techniques, “top-to-bottom” and “bottom-up,” have reported favorable complication rates and satisfaction. Different injection planes such as subcutaneous injection, fat layer injection, and supraperiosteal combined with subcutaneous injection have also been extensively studied. The first two techniques have reported erythema incidence rates of 5% (14/280) and 2.1% (2/96), respectively, which are slightly higher than more traditional injection protocols. And the “critical site injections,” which allow for a more flexible order of injections through the detailed design of the different points, may become a more streamlined and standardized injection protocol option.

### Complication

Table 4 gives the complication rates reported in the included literature.

**Table 4:** Complication rate and treatment

Complication			No. of AEs	Rate^*^		Treatment
Intraoperative complications		Bleeding	612	8.34%	10.03%	Mostly no treatment required, a few need hyaluronidase depending on the situation.
		Arterial occlusion	24	0.33%		
		Immediate bruising	100	1.36%		
Postprocedural complications	Mild	Hematoma/Bruising	148	2.02%	13.35%	With the "wait and see" approach, the majority of symptoms are observed to subside in 2 days. And specific treatments include icing, massage, hot moist compresses, and antibiotics.
		Asymmetry	21	0.29%		
		Mild edema	683	9.31%		
		Enlargement at the nasal tip	2	0.03%		
		Transient redness of the skin	2	0.03%		
		Mild pain	49	0.67%		
		Headache	8	0.11%		
		Erythema	59	0.80%		
		Itchiness	8	0.11%		
	Moderate	Late erythema and late edema	58	0.79%	0.86%	Antibiotics, steroids, and hyaluronidase are often utilized.
		Infection	5	0.07%		
	Severe	Vascular occlusion	4	0.05%	0.33%	
		Vision loss	10	0.14%		
		Skin necrosis	5	0.07%		
		Cerebral infarction	3	0.04%		
		Hypersensitivity	1	0.01%		
		Granuloma	1	0.01%		
Total			1803	24.57%		

*Total number of patients is 7339 (as in Table 1)

The overall complication rate was 24.57% in 7339 patients. Most of the complications that account for the high complication rate are slight intraoperative and postoperative complications that do not require active treatment. Among these, the most frequent events were intraoperative bleeding, postoperative hematoma/bruising, and mild edema. The rate of moderate to severe complications was only 1.19%, with severe vascular complications occurring in as few as 0.3%. Notably, the 20 patients in the case report reported 18 complications (Supplementary Table 3), essentially serious complications. The incidence of serious complications reported in included studies other than case reports was less than 0.1%.

## Discussion

In recent years, non-surgical rhinoplasty has become a popular treatment choice in the field of esthetics. Based on the results of literature screening, clinicians have proposed a number of different injection options, and a series of studies regarding safety and efficacy have been made. Almost all studies reported very satisfactory results: lower rates of adverse events and higher satisfaction than surgical rhinoplasty. This may be related to the lower cost of liquid rhinoplasty and the reversibility with hyaluronidase. We believe that patients can make the decision to undergo non-surgical rhinoplasty with less psychological burden, compared to surgical rhinoplasty. Thus, it can be an option for patients with smaller deformities and those who wish to preoperatively evaluate the results of surgical rhinoplasty.

Different injection protocols are differentiated by the patient's condition, in addition to complications, satisfaction, ethnicity, and clinician's preferences, all of which influence the choice of protocol. Based on the idea that injections along the nasal midline and supraperiosteal layers minimize the risk of complications, the bottom-up [48] and top-to-bottom [4, 14] approaches are the most widely recognized injection options and are proven to be suitable for both Caucasians and Asians. The difference between the two is that clinicians who favor the bottom-up injection approach believe that more serious complications, such as blindness and cerebral infarction, are attributable to manipulation at the nasal root or between the eyebrows. Exactly, bottom-up injections minimize the amount of fill in this area. Erythema, bruising, and swelling are commonly reported complications in the bottom-up and top-to-bottom approaches. Serious vascular complications are rare. Blindness and skin necrosis occurred in less than 1‰ [26].

A more innovative injection method is the multi-plane injection technique, all of its patients included in this article received injections after 2016 [23]. Injecting at a more superficial location results in a better appearance with less filler. The downside, however, is a slight increase in the rate of complications. Though no serious complications were reported, this type of innovative technique has not been tested in a large number of practices, which affects the comprehensiveness of the results. Considering the vascular distribution, it cannot be neglected that innovative injection approaches carry a higher risk of vascular complications than traditional approaches. It is worth mentioning that it did not obtain more outstanding satisfaction, compared to other approaches, which may be related to the appearance of mild adverse events.

Another trend is toward more process-oriented, standardized injection protocols, possibly through the “critical site injections.” Based on a detailed delineation of the injection area, it offers the possibility of enhancing patient safety, improving therapeutic consistency, and designing more specific injection programs for different indications. One of the included papers [7] reported a high (10%; 2/20) incidence of hematoma requiring no active treatment, which we attribute to the smaller sample size and more needle entry points. More safety tests and Asians’ effectiveness studies are expected.

The complication rate as a whole, without distinguishing between injection techniques, is not actually low enough to sleep soundly. Table 3 indicates an overall postoperative complication rate of 14.5%. Most of these resolved rapidly based on a “wait and see” approach within about 2 days. Specific treatments include icing for bruising [49], massage for asymmetry [22], hot moist compresses for hematomas [22], and oral corticosteroids [24] (e.g., triamcinolone acetonide [26]) for advanced edema. In case of serious complications such as infection or skin necrosis, administer antibiotics, and steroid (e.g., dexamethasone) [13] in the first instance and consider dissolving the filler with hyaluronidase [26]. Severe asymmetries are also treated with hyaluronidase applied according to the patient's needs [49]. Skin necrosis can be restored with the prompt use of hyaluronidase, but a considerable portion of vision loss is fully or partially irreversible [11, 36, 40]. Timely use of hyaluronidase, however, can still have a positive effect [13, 50].

The limitation of this study is that there may be the presence of multiple complication events occurring in a single patient in the statistics of complications, and therefore, the calculated value of complications can be considered as event rates. Additionally, a handful of injection-related factors were not included in the analysis. Further research could consider an in-depth analysis starting with factors such as cannula versus sharp needle, injection speed, aspiration, and use of ultrasound guidance.

## Conclusion

Rhinoplasty injections still have a non-negligible complication rate. Especially in clinics where the doctor's qualifications and the quality of the fillers are unknown, there are serious complications. An effective way to improve safety and satisfaction may be process-oriented injection techniques.

## Supplementary Information

Below is the link to the electronic supplementary material.

Supplementary file1 (DOCX 34 KB)
