# Supplementary material for: Advances of Hyaluronic Acid Nasal Injection Techniques and Complications: A Systematic Review
Source: Aesthetic Plast Surg. 2025 Sep 2;49(23):6675–86. doi: 10.1007/s00266-025-05194-z (PMC12738657; doi:10.1007/s00266-025-05194-z)
Supplement: Supplementary file 1 — Supplementary file1 (DOCX 34 KB) [file 266_2025_5194_MOESM1_ESM.docx]

**Supplemental Appendix**

**Advances of hyaluronic acid nasal injection techniques and Complications: a systematic review**

**Contents**

**Supplementary Tables**

**Supplementary Table 1. Excluded-Studies appendix** 2

**Supplementary Table 2. Demerit points for risk of bias of the included studies** 3

**Supplementary Table 3. Complications reported in case reports** 5

**Supplementary Table 1. Excluded-Studies appendix**

| Author | Year | Title | Type of work | Excluded reasons |
| --- | --- | --- | --- | --- |
| Bravo BSF et al. | 2024 | Nasal filling guided by high frequency ultrasound: Reducing risks | prospective | Overfocus on vascular-related complications and lack of information on other complications |
| Calvisi, Lucia et al. | 2022 | Nonsurgical reshaping of the nose, chin, and jawline: A retrospective analysis using experience-based eligibility criteria | retrospective | Unable to distinguish whether the complication is nasal or elsewhere |
| Han, Xuefeng et al. | 2015 | Multiplane hyaluronic acid (EME) in female Chinese rhinoplasty using blunt and sharp needle technique | retrospective | Lack of reporting of mild complications |
| Helmy, Yasser | 2018 | Non-surgical rhinoplasty using filler, Botox, and thread remodeling: Retro analysis of 332 cases | retrospective | Inadequate description of injection techniques |
| Nguyen, T.A. et al. | 2020 | Specific complications associated with non-surgical rhinoplasty | review | Inconsistency in type of work |
| Robati, Reza M et al. | 2018 | The Risk of Skin Necrosis Following Hyaluronic Acid Filler Injection in Patients With a History of Cosmetic Rhinoplasty | retrospective | Inadequate description of injection techniques |
| Alessio Redaelli | 2008 | Medical rhinoplasty with hyaluronic acid and botulinum toxin A: a very simple and quite effective technique | retrospective | Involving fillers other than hyaluronic acid |
| F Braccini et al. | 2008 | Medical rhinoplasty: rationale for atraumatic nasal modelling using botulinum toxin and fillers | retrospective | Involving fillers other than hyaluronic acid |
| HJ Zhang et al. | 2017 | Clinical application and observation of injectable modified sodium hyaluronate gel filler for facial cosmetic surgery | retrospective | Unable to distinguish whether the complication is nasal or elsewhere |

**Supplementary Table 2. Demerit points for risk of bias of the included studies**

| Study | Rating explanation |
| --- | --- |
| ^*^Li, Dong; 2022 | Selection bias: The allocation sequence was hidden and randomized, but the baseline was slightly uneven between groups. |
|  | Performance bias: Not sure about blinding, but results are relatively unaffected by blinding |
|  | Detecting bias: Blinding has little effect on outcome assessment |
|  | Attrition bias: Between-group differences in the proportion of missing data. |
|  | Reporting bias: All pre-asserted endings of concern have been reported. |
| ^*^Wang, Xiaojun; 2022 | Selection bias: The allocation sequence was hidden and randomized. |
|  | Performance bias: Lack of blinding |
|  | Detecting bias: Conducted by a blinded, independent evaluator |
|  | Attrition bias: Existence of missed visits during follow-up |
|  | Reporting bias: All pre-asserted endings of concern have been reported. |
| Liapakis, I. E.; 2013 | High proportion of subjective indicators or the main measurement is subjective (-1); No consideration of confounding factors (-1) |
| Segreto, Francesco; 2019 | High proportion of subjective indicators or the main measurement is subjective (-1); No consideration of confounding factors (-1) |
| Turk, Cemre Busra; 2024 | High proportion of subjective indicators or the main measurement is subjective (-1); No consideration of confounding factors (-1) |
| Yordanov, Y P; 2019 | High proportion of subjective indicators or the main measurement is subjective (-1); No consideration of confounding factors (-1) |
| Romeo F; 2023 | High proportion of subjective indicators or the main measurement is subjective (-1); No consideration of confounding factors (-1) |
| Jung GS; 2019 | High proportion of subjective indicators or the main measurement is subjective (-1); No consideration of confounding factors (-1); Less than six months of follow-up with possible complications not observed (-1) |
| Josipovic LN; 2021 | High proportion of subjective indicators or the main measurement is subjective (-1); No consideration of confounding factors (-1) |
| Bektas, Gamze; 2020 | High proportion of subjective indicators or the main measurement is subjective (-1); No consideration of confounding factors (-1) |
| Bertossi, Dario; 2021 | High proportion of subjective indicators or the main measurement is subjective (-1); No consideration of confounding factors (-1) |
| Chen, B; 2020 | High proportion of subjective indicators or the main measurement is subjective (-1); No consideration of confounding factors (-1) |
| Giammarioli, Giulio; 2023 | High proportion of subjective indicators or the main measurement is subjective (-1); No consideration of confounding factors (-1) |
| Harb, A; 2024 | High proportion of subjective indicators or the main measurement is subjective (-1); No consideration of confounding factors (-1); Less than six months of follow-up with possible complications not observed (-1); More than 10% of patients lost to follow-up (-1) |
| Harb, A; 2020 | High proportion of subjective indicators or the main measurement is subjective (-1); No consideration of confounding factors (-1); Less than six months of follow-up with possible complications not observed (-1); More than 10% of patients lost to follow-up (-1) |
| Jalali, Arash；2024 | High proportion of subjective indicators or the main measurement is subjective (-1); No consideration of confounding factors (-1); Less than six months of follow-up with possible complications not observed (-1) |
| Liew, Steven; 2016 | High proportion of subjective indicators or the main measurement is subjective (-1); No consideration of confounding factors (-1) |
| Nikolis, Andreas; 2023 | High proportion of subjective indicators or the main measurement is subjective (-1); No consideration of confounding factors (-1) |
| Rauso, Raffaele; 2017 | High proportion of subjective indicators or the main measurement is subjective (-1); No consideration of confounding factors (-1); Less than six months of follow-up with possible complications not observed (-1) |
| Santorelli, Adriano; 2020 | High proportion of subjective indicators or the main measurement is subjective (-1); No consideration of confounding factors (-1) |

**^*^**These studies are RCTs. The quality evaluation for RCTs used the Cochrane risk of bias tool. Other non-randomized trials used the NOS scale.

**Supplementary Table 3. Complications reported in case reports**

| Complication | n |
| --- | --- |
| Headache | **1** |
| Hypersensitivity | **1** |
| Vascular occlusion | **2** |
| Granulomas | **1** |
| Cerebral infarction | **3** |
| Vision loss | **8** |
| Skin necrosis | **2** |
| Total | **18** |
